# Comprehensive evaluation of ten deformable image registration algorithms for contour propagation between CT and cone-beam CT images in adaptive head & neck radiotherapy

**DOI:** 10.1371/journal.pone.0175906

**Published:** 2017-04-17

**Authors:** Xin Li, Yuyu Zhang, Yinghua Shi, Shuyu Wu, Yang Xiao, Xuejun Gu, Xin Zhen, Linghong Zhou

**Affiliations:** 1 Department of Biomedical Engineering, Southern Medical University, Guangzhou, Guangdong, China; 2 Department of Radiotherapy Oncology, The First Hospital of Jilin University, Changchun, Jilin, China; 3 Department of Radiotherapy Oncology, The University of Texas, Southwestern Medical Center, Dallas, Texas, United States of America; North Shore Long Island Jewish Health System, UNITED STATES

## Abstract

Deformable image registration (DIR) is a critical technic in adaptive radiotherapy (ART) for propagating contours between planning computerized tomography (CT) images and treatment CT/cone-beam CT (CBCT) images to account for organ deformation for treatment re-planning. To validate the ability and accuracy of DIR algorithms in organ at risk (OAR) contour mapping, ten intensity-based DIR strategies, which were classified into four categories—optical flow-based, demons-based, level-set-based and spline-based—were tested on planning CT and fractional CBCT images acquired from twenty-one head & neck (H&N) cancer patients who underwent 6~7-week intensity-modulated radiation therapy (IMRT). Three similarity metrics, i.e., the Dice similarity coefficient (DSC), the percentage error (PE) and the Hausdorff distance (HD), were employed to measure the agreement between the propagated contours and the physician-delineated ground truths of four OARs, including the vertebra (VTB), the vertebral foramen (VF), the parotid gland (PG) and the submandibular gland (SMG). It was found that the evaluated DIRs in this work did not necessarily outperform rigid registration. DIR performed better for bony structures than soft-tissue organs, and the DIR performance tended to vary for different ROIs with different degrees of deformation as the treatment proceeded. Generally, the optical flow-based DIR performed best, while the demons-based DIR usually ranked last except for a modified demons-based DISC used for CT-CBCT DIR. These experimental results suggest that the choice of a specific DIR algorithm depends on the image modality, anatomic site, magnitude of deformation and application. Therefore, careful examinations and modifications are required before accepting the auto-propagated contours, especially for automatic re-planning ART systems.

## Introduction

Intensity-modulated radiation therapy (IMRT) plays a critical role in the management of head and neck (H&N) cancer patients [1]. IMRT can maximize tumor coverage and/or sparing of organs at risk (OARs) by generating a steep dose gradient and, thus, leads to a potential increase in the therapeutic index [2, 3]. However, it does not take into account fractional anatomical changes, such as tumor shrinkage, nodal/glandular volume change, weight loss and geometric variations, etc., during a typical 6~7-week treatment course [4, 5]. Adaptive radiation therapy (ART) [6] is a possible solution to overcome these limitations by adapting temporal changes in anatomy with daily imaging in the radiotherapy process [7–9]. In a typical online ART process, a cone-beam computed tomography (CBCT) scan is usually performed to obtain three-dimensional patient information prior to treatment, and a deformable image registration (DIR) technique is utilized to establish the correspondence between voxels in the planning computed tomography (CT) and CBCT scans for 1) propagating the contoured region of interest (ROI) from one image to another [10, 11] and 2) deforming the re-planned dose maps to integrate the accumulated dose administered to the patient [12, 13].

Effective and reliable ART relies upon accurate DIR-propagated ROIs, and thus, it is necessary to verify the accuracy of the available DIR algorithms for use in a clinical setting. There are a variety of studies that have assessed the quality of CT-CT DIR algorithms in H&N cancer patients; for instance, Castadot *et al*. [14] compared twelve voxel-based DIR strategies in ROI propagation performance using expert physician-drawn contours as benchmarks. Hardcastle *et al*. [15] investigated the clinical utility of two DIR algorithms for ROI propagation using CT image data from five institutions. They reported good anatomical agreements for OARs but claimed that the propagated target structures need to be thoroughly reviewed before clinically use. Mohamed *et al*. [16] developed a quality assurance workflow for the quantitative assessment of four different DIR techniques used for H&N radiation therapy-simulation CT with diagnostic CT co-registration. A total of 2720 anatomic and 50 tumor/nodal ROIs were used for validation, and improved performance with DIR over rigid registration was observed. Limited studies, however, have focused on the comparison of CT-CBCT DIR algorithms in assessing H&N patient data. Though a number of CT-CBCT DIR methods for different clinical sites have been developed [11, 17–23], it is acknowledged that some DIR algorithms may be more suitable for specific anatomies and image modalities due to the mathematical basis of the algorithms [24]. In this sense, it is beneficial to assess the clinical acceptability of the available DIR algorithms to reduce the time and resources required for contour review and correction in the CBCT-based ART process [15].

The aim of this study was to develop a strict and objective methodology to compare different DIR strategies for contour propagation from the planning CT (PCT) to the treatment CBCT of H&N cancer patients in an adaptive radiotherapy setting.

## Materials and methods

### Patient cohort

Seven female and fourteen male patients diagnosed as nasopharyngeal cancer (NPC) were included in this study. Details of the evaluated patients are listed in Table 1. All patients were treated by IMRT with fractional CBCT for setup error correction. Immobilization was necessary and was achieved by a customized thermoplastic mask during the acquisition of the PCT and CBCT. The PCT images were reconstructed with a resolution of 1.60×1.60×5 mm^3^ along the x, y and z directions, respectively. The CBCT images at a resolution of 0.51×0.51×1.99 mm^3^ were acquired before the treatment. The size of axial images for both PCT and CBCT was 512×512. There were 89 slices in each CBCT scan, and the number of slices in the PCT images ranged from 60 to 130. A total of 21 PCTs and 129 CBCTs were utilized in this study.

**Table 1:** Patients’ characteristics.

Patient #	Gender	Age	Histology	TNM stage	Concurrent CBCT
1	F	44	NPC	T4N1M0 IVA	Yes
2	F	45	NPC	T2N3M0 IVA	Yes
3	F	48	NPC	T3N3M0 IVA	Yes
4	F	59	NPC	T2N2M0 III	Yes
5	M	41	NPC	T1N2M0 IVA	Yes
6	F	67	NPC	T4N3M1 IVB	Yes
7	M	64	NPC	T3N1M0 III	Yes
8	M	50	NPC	T4N1M0 IVA	Yes
9	M	39	NPC	T4N2M0 IVA	Yes
10	M	55	NPC	T2N3M0 IVA	Yes
11	M	39	NPC	T3N2M0 III	Yes
12	M	60	NPC	T2N0M0 II	Yes
13	F	55	NPC	T2N2M0 IVA	Yes
14	M	63	NPC	T2N1M0 IVA	Yes
15	M	46	NPC	T1N2M0 III	Yes
16	F	48	NPC	T2N2M0 III	Yes
17	M	52	NPC	T4N2M0 IVA	Yes
18	M	48	NPC	T4N1M0 IVA	Yes
19	M	37	NPC	T4N1aM0 IVB	Yes
20	M	40	NPC	T4N1M0 IVA	Yes
21	M	67	NPC	T4N1M0 IVA	Yes

In this study, all the CT and CBCT images were retrospectively collected and anonymized for clinical research purposes. Our study did not involve new CT or CBCT scans of patients and, therefore, did not require approval of the local ethics committee. All patients provided written informed consent for their images to be used for research purposes and to be published.

### Delineation of ROIs

OARs including the parotid gland (PG), the submandibular gland (SMG), the cervical vertebra (VTB) and the vertebral foramen (VF), on both PCT and CBCT were manually delineated by an experienced physician using a commercial treatment planning system (TPS, Eclipse 10.0, Varian) and double checked by this same physician three months later to ease intra-observer variations. The contours delineated on the PCT served as the moving contours being deformed to the reference fractional CBCT domain, while the contours delineated on the CBCT images served as the ground truths for contour propagation accuracy validation in this study. We chose the deformable organ PG and SMG instead of the gross target volume (GTV) for this evaluation study for two reasons. First, many pilot studies have revealed that large volume shrinkage or deformation can be seen in the PG and the SMG during H&N radiation treatment [4, 25–36], and thus, contour propagation of the PG and SMG onto the fractional CBCT anatomy is usually necessary for treatment re-planning or planning optimization. Second, GTV in CBCT images is difficult to identify due to the low soft tissue contrast and severe image artifacts present in CBCT. Using GTV with erroneous contouring in CBCT images as the ground truth would bias the evaluation result.

### Image preprocessing

Before performing the rigid and deformable registration, all the images were pre-processed using an open source software 3D Slicer (version 4.3.1, http://www.slicer.org) [37]. Unnecessary image content such as the treatment table and the thermoplastic mask were manually segmented out from all the images. Because the superior-inferior (S-I) patient volume range in the PCT was generally larger than that in the CBCT, in order to unify the image size and save computational time, the PCT was cropped and re-sampled to match the dimension and resolution of the CBCT after rigid registration, which was also done in 3D Slicer. Hence, the image resolution for both the PCT and CBCT images after rigid registration was 256×256×89 for all the evaluated cases. The regions of interest (103 mm×103 mm×80 mm for PG; 79 mm×79 mm×65 mm for SMG) that encompass each PG and SMG were extracted for evaluation. For VTB and VF, because the imaged anatomies were not consistent in PCT and CBCT, we extracted the same anatomic regions in PCT and CBCT for evaluation to ensure anatomy consistency.

### Evaluated DIR algorithms

In this work, ten intensity-based deformable image algorithms were evaluated. These DIRs methods were classified into four categories: optical flow based, demons based, level-set based and spline based. Representative algorithms in each category were chosen for validation (listed in Table 2). For the ten selected algorithms, eight (HS [38], HSLK [39], FFD [40], OD [41], MD [42], SFD [43], DFD [44] and LS [45]) were implemented using an open source DIR toolkit-the DIRART [46]. The BSpline method was performed by another open source DIR package, Elastix [47], and the DISC was an in-house GPU-based DIR algorithm developed previously by our group [19].

**Table 2:** Overview of Ten DIR Algorithms.

Class	Algorithm	Toolkit/Implementation Environment	Acronym
Optical flow	Original Horn-Schunck method	DIRART/Matlab	HS
	Combined Horn-Schunck and Lucas-Kanade method	DIRART/Matlab	HSLK
	Free-Form Deformation method	DIRART/Matlab	FFD
Demons	Original Demons method	DIRART/Matlab	OD
	Modified Demons method	DIRART/Matlab	MD
	Symmetric Force Demons method	DIRART/Matlab	SFD
	Double Force Demons method	DIRART/Matlab	DFD
	Deformation with Intensity Simultaneously Corrected	CUDA/GPU	DISC
Level-set	Original Level-Set Motion method	DIRART/Matlab	LS
Spline	B-Spline method	Elastix/C++	BSpline

### Grouping and staging

We classified the ROIs into two structural groups, i.e., bony structures including the VTB and the VF, and the soft-tissue structures including the PG and the SMG. Considering that the volume of the PG or the SMG may vary irregularly during the 6~7-week treatment course, we partitioned the entire treatment into six different stages; each stage included five consecutive treatment fractions. ROI volume variations in each stage and its relationship with DIR performance were analyzed.

### ROI volume variation

In this study, the right and left parotid or submandibular glands were treated as independent structures (termed as rPG, lPG, rSMG and lSMG, respectively) in light of the fact that they are indeed anatomically isolated (though functioning similarly) and respond separately to incident radiation beams. The relative volume change of each structure in each patient over the entire treatment course was analyzed using linear regression analyses.

### Contour propagations

Deformation of ROI contours was performed similar to in Castadot *et al*. [14]. First, all ROI contours on PCT and CBCT were converted to a binary mask where the voxels inside and outside the contours were labeled as one and zero, respectively. After DIR, the resulting deformation vector field (DVF) was used to transform the ROI mask on the PCT and to yield a deformed mask with continuous values between 0 and 1. A threshold of 0.5 was empirically chosen to binarize the deformed mask whose contours were then compared with the ground truths.

### Evaluation metrics

To quantitatively evaluate the agreement between the deformed OARs and the reference OARs, three similarity metrics were used: the Dice similarity coefficient (DSC), the percentage error (PE) and the Hausdorff distance (HD). Given the ground truth reference region *A*, the deformed region *B*, and their corresponding boundary point sets $\vec{A}=\{a_{1},\cdots ,a_{p}\}$ and $\vec{B}=\{b_{1},\cdots ,b_{q}\}$, the DSC is defined as *DSC* = 2(*A* ∩ *B*)/(*A* + *B*), which ranges from 0 to 1, corresponding to the worst and the best segmentations, respectively. The PE is defined as *PE* = (*A* ∪ *B* − *A* ∩ *B*)/*A*, with 0 representing the best segmentation. The HD is defined as $HD=max\left(h(\vec{A},\vec{B}),h(\vec{B},\vec{A})\right)$, where $h(\vec{A},\vec{B})=\max_{a\in \vec{A}}\left(\min_{b\in \vec{B}}\left(\left\|a-b\right\|\right)\right)$ and ‖⋅‖ are the *L*_2_ norms on the points of $\vec{A}$ and $\vec{B}$.

### Statistical analysis

Descriptive statistics were calculated to characterize the volume changes of the PG and the SMG during the treatment course, and linear regression analyses were used to compute the slopes of such changes in the relative volume for each structure in each patient.

Independent samples Kruskal-Wallis (K-W) test was adopted for performance comparisons among all algorithms, for performance variations of the same DIR on four different ROIs and for performance variations of different DIRs on the same ROI. Inter-group comparisons were performed with Mann-Whitney tests. Within each stage, K-W tests and one-way analysis of variance (ANOVA) were conducted for non-parametric and parametric data, respectively, to analyze DIR performance difference.

All statistical analyses were implemented using SPSS 19.0 software (SPSS Inc., Chicago, IL), and the statistical significance level was set at *p* = 0.05. For multiple comparisons, the p-value was adjusted accordingly using the Bonferroni correction method for individual comparison tests in SPSS.

## Results

### Volume changes

**Fig 1:**
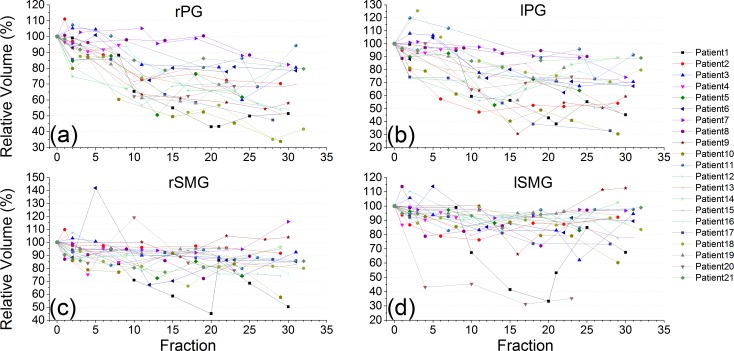
Time trend of the relative volume change for the rPG (a), lPG (b), rSMG (c) and lSMG (d) over the course of the treatment.

As shown in Fig 1, the volumes of both the PG and the SMG reduced over the course of the treatment, and the left and right parts of the PG or the SMG demonstrated similar volume reductions. However, the volume of the PG decreased about two times faster than that of the SMG. The mean value, standard deviation (SD), 95% confidence interval (95% CI) and interquartile range (IQR) of the slopes of the relative volume change for each structure are given in Table 3.

**Table 3:** The mean, standard deviation (SD), 95% confidence interval (95% CI) and interquartile range (IQR) of the slopes of relative volume changes for each structure.

Structure	Mean (%/td^*^)	SD	95% CI	IQR
rPG	-1.208	0.540	[-1.454;-0.962]	0.898
lPG	-1.170	0.608	[-1.447;-0.894]	0.629
rSMG	-0.546	0.369	[-0.714;-0.378]	0.495
lSMG	-0.471	0.600	[-0.745;-0.198]	0.656

^*^ %/td: percentage per treatment day

### Algorithm comparisons

#### Comparison for all ROIs

**Fig 2:**
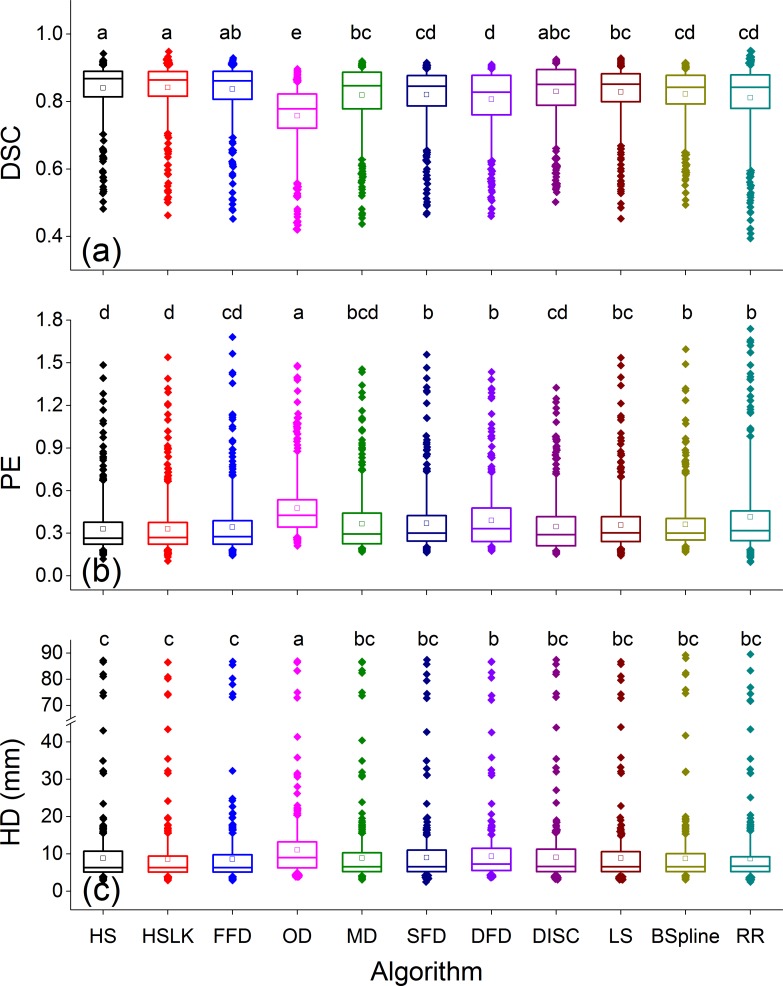
Boxplots of DSC (a), PE (b) and HD (c) by different DIR algorithms for all structures in all treatment stages. The boxes run from the 25th to 75th percentile; the two ends of the whiskers represent the 5% and 95% percentiles of the data, the horizontal line and the square in the box represent the median and mean values, respectively. The diamonds represent outliers. The letters above each box indicate whether a statistically significant difference exists between any two DIRs. No common letter between any two algorithms indicates that the two DIRs are significantly different. RR is an abbreviation for rigid registration.

Fig 2 shows that comparisons among all algorithms in terms of DSC, PE and HD in all structures. We made the following observations: 1) Rigid registration performed worse than HS, HSLK and FFD (*p*<0.001, *N* = 508) in terms of DSC and PE. However, rigid registration performed better than OD on all metrics (*p*<0.001, *N* = 508). 2) OD was the worst DIR algorithm and performed significantly worse than the other DIRs in three metrics (*p*<0.001, *N* = 508). 3) The optical flow-based algorithms performed best, and the DISC ranked second among all algorithms.

#### Inter-group and intra-group comparisons

**Fig 3:**
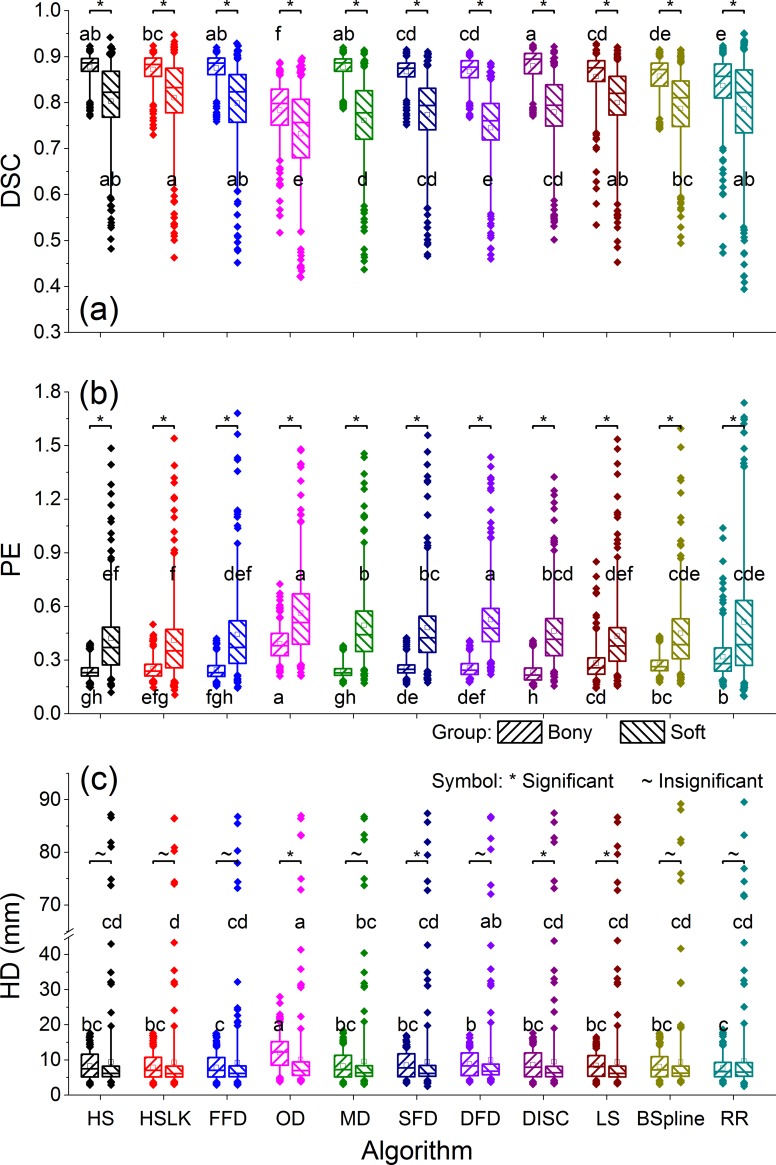
Boxplots of DSC (a), PE (b) and HD (c) by different DIR algorithms for the bony and soft-tissue groups in all treatment stages. The meanings of the symbols in this figure are the same as in Fig 2.

The DIR performance comparison between the bony group and the soft-tissue group is depicted in Fig 3. The Mann-Whitney analysis showed that, for all registration methods, DIR performance in the bony group was better than that in the soft–tissue group in terms of DSC and PE (*p*<0.001, *N* = 254). In addition, the precision of each algorithm was comparatively high in the bony group where smaller standard deviations of all metrics were found.

In the bony group, all DIRs except for BSpline and OD were better than rigid registration in terms of DSC and PE (DFD: *p* = 0.002 for DSC; LS: *p* = 0.019 for DSC and *p* = 0.029 for PE; SFD: *p* = 0.022 for DSC; others: *p*<0.001 for DSC and PE, *N* = 254), while in the soft-tissue group, DIRs were comparable to or even worse than the rigid algorithm. DISC and HS/HSLK were the best two DIRs in the bony and soft group, respectively, while the OD performed significantly worse than the other DIR algorithms for all metrics (bony: *p*<0.001; soft-tissue: MD: *p* = 0.005 for DSC, *p* = 0.025 for PE, *p* = 0.013 for HD; others: *p*<0.001, *N* = 254).

#### Comparisons across ROIs

**Fig 4:**
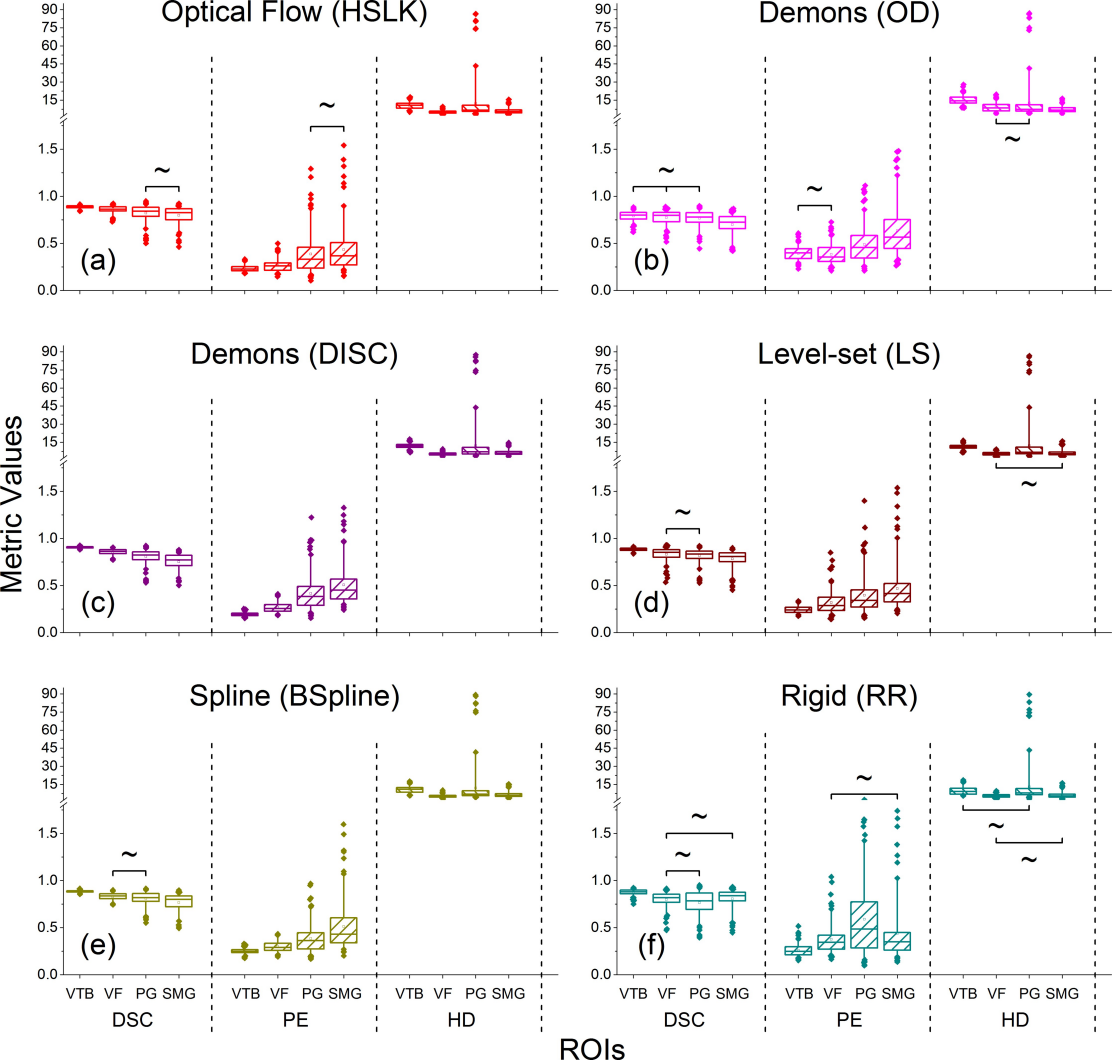
Boxplots of comparisons of performance on different ROIs for representative DIR algorithms, including the HSLK (optical flow-based), OD/DISC (demons-based), LS (level-set-based), BSpline (spline-based) and rigid registration (RR). For each subgraph, no significant difference in pairwise comparisons is marked with “~”, otherwise a statistically significant difference exists. The meanings of each box in this figure are the same as in Fig 2.

Fig 4 illustrates the performance of the different DIR algorithms on individual ROIs. We made the following observations: 1) For a same DIR, the performance varied significantly among different ROIs (*p*<0.05, *N* = 127). 2) For the DSC and PE metrics, most DIR algorithms (e.g., DISC (*p*<0.001), MD (*p*<0.001) and FFD (*p* = 0.007 and *p* = 0.028) in the VTB for DSC and PE; all DIRs (LS: *p* = 0.001 and *p* = 0.006, others: *p*<0.001) but OD and BSpline in the VF for DSC and PE; HSLK (*p* = 0.004) in the PG for PE, *N* = 127) were superior to rigid registration in the VTB, VF and PG; however, rigid registration performed better than some DIRs, such as OD, DFD and MD (MD: *p* = 0.018 for HD; others: *p*<0.001 for all metrics, *N* = 127) in SMG. 3) For each ROI, DIR performance varied significantly across different DIR methods for all metrics. Generally, the optical flow-based algorithms performed comparatively better in the VF, PG and SMG. The demons-based algorithms had a comparatively worse performance in all ROIs, among which OD performed significantly worse than all other algorithms including the rigid method for all assessed metrics (*p*<0.001, *N* = 127). 4) DISC, a modified demons-based algorithm, was comparable to the optical flow-based methods.

#### Comparisons across ROIs and stages

**Fig 5:**
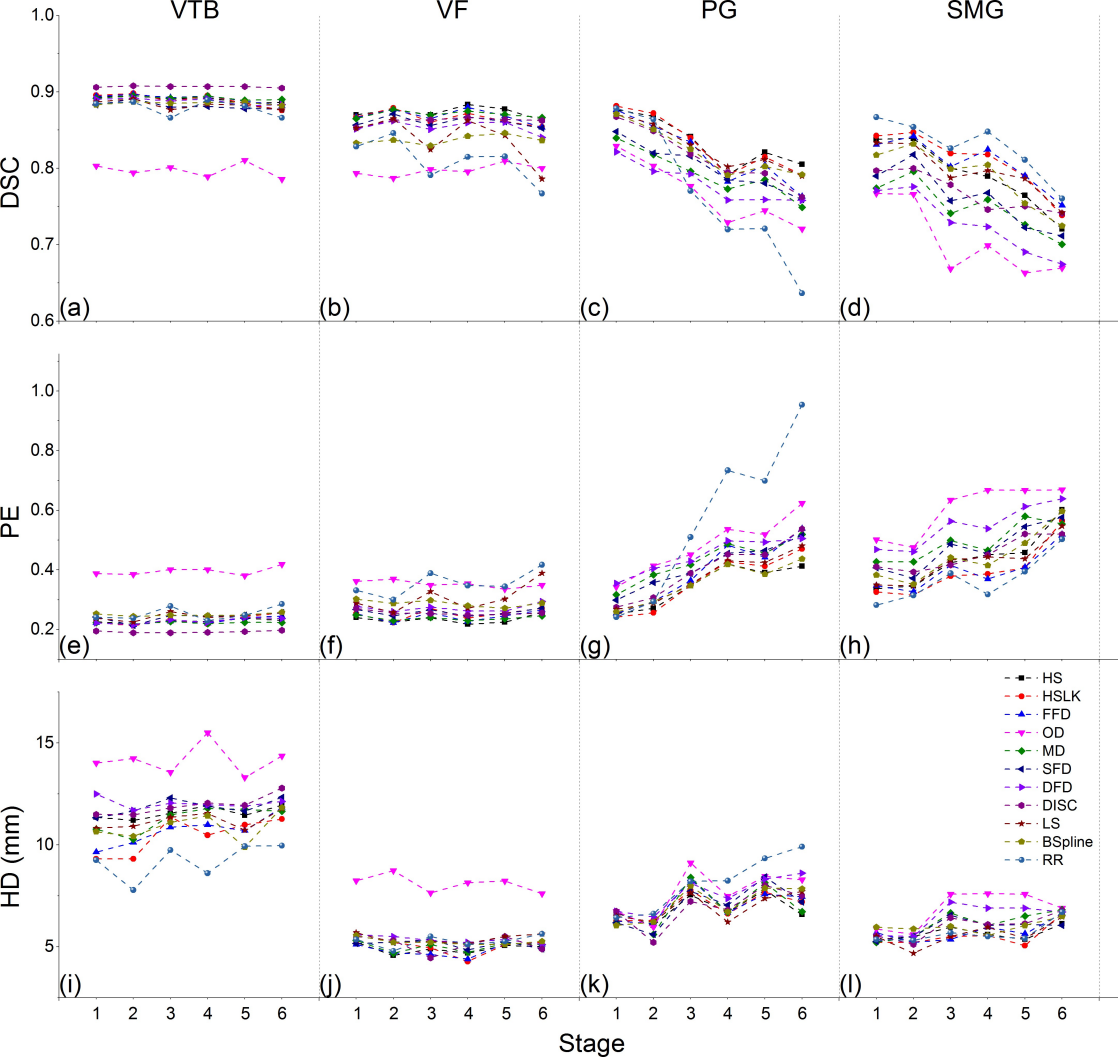
Comparisons across ROIs and stages for different registration algorithms. In each subgraph, the horizontal and vertical axes represent stages ranging from 1 to 6 and the metric values, respectively. Eleven algorithms are marked by different symbols and colors.

Fig 5 depicts the comparison results of all registration methods of each stage for each ROI and the performance variations during the treatment course. We found that 1) For all metrics, the performances of all registration methods in the bony group were more stable than those in the soft-tissue group. For rigid registration, performance in the bony group was comparable with the soft-tissue group in early stages (stage 1 and 2), but better in the subsequent stages. 2) OD performed worst among all methods. 3) Staging comparisons of each registration method varied with ROI.

For bony structures, the performance of all algorithms remained stable over the treatment. In all stages, rigid registration performed worse than some DIR in terms of DSC and PE, such as DISC in the VTB (stage 2: *p* = 0.002 for DSC and PE; stage 4: *p* = 0.008 for DSC and *p* = 0.005 for PE; others: *p*<0.001) and HS in the VF (stage 1: *p* = 0.142 for DSC and *p* = 0.062 for PE; stage 2: *p* = 0.004 for DSC; stage 4: *p* = 0.018 for DSC and *p* = 0.003 for PE; stage 6: *p* = 0.021 for DSC and *p* = 0.008 for PE; others: *p*<0.001). Otherwise, the performance of the rigid method was comparable to that of the other DIRs except for OD. In all stages, two demons-based DIRs (DISC and MD) and two optical flow-based DIRs (HS and FFD) performed best in the VTB and in the VF, respectively.

For both the PG and the SMG, the performance of all registration methods on DSC and PE deteriorated as the stage increased, while rigid registration dropped more quickly than DIRs, especially in the PG. In all stages, the optical flow-based algorithms performed best, followed by the level-set-based or spline-based methods, and the demons-based methods ranked last.

## Discussion

In this study, we extensively compared the ability of different DIR strategies for OAR contour propagation between CT and CBCT images. Ten DIR algorithms, including HS, HSLK, FFD, OD, MD, SFD, DFD, DISC, LS and BSpline, were validated employing planning CT and treatment CBCT images that were retrospectively acquired from twenty-one NPC patients.

Regarding OAR volume change throughout the treatment in NPC patients, we observed similar volume reductions in the PG and the SMG as previously published [4, 25–36]. For instance, Robar *et al*. [25] found that the left and right parotid shrank by 4.7% and 5.0% per week. Comparable results were revealed by Huang *et al*. [35] that the volume of the left and right parotid glands reduced by 7.9%/week and 7.8%/week, respectively. Similarly, Castadot *et al*. [30] reported that the volume reduction of the ipsilateral and contralateral parotid were, respectively, 0.9%/day and 1.0%/day, and 1.5%/day and 1.3%/day for the SMG. In a longitudinal study, Wang *et al*. [29] investigated 82 patients and found that, after receiving three weeks of treatment, the average volume loss at the end of treatment and at two months after treatment were 20.01%, 26.93% and 27.21% for the PG and 11.49%, 16.76% and 16.29% for the SMG, implying that the PG was more sensitive to radiation and would suffer larger volume loss than the SMG.

Before applying the DIR, the treatment table and the thermoplastic mask in all planning CT images and treatment CBCT images were carefully segmented and removed by an experienced physician, leaving only the patient volume in the images, to avoid registration bias induced by such exotic apparatuses. To account for the relationship between DIR performance and volume variation, we compared OARs with changing and constant volumes. Furthermore, we divided the treatment course into different stages for DIR performance analysis. The reasons were threefold, as follows: First, we wanted to determine whether the evaluated DIR algorithms are stable across the entire treatment course, given that some OARs might deform irregularly in different treatment stages. Second, the performance discrepancy between different DIR methods might be related to the different scales of OAR deformation, which might occur in different treatment stages. Third, it would be interesting to know whether DIRs are superior to rigid registration during the entire treatment course.

In many published evaluations regarding DIR performance in H&N patients, DIR is usually believed to be more accurate than rigid registration in OAR contour propagation. For example, Castadot *et al*. [14] compared 12 DIR methods (including the demons-based, level-set-based and fast free-form-based DIRs) by evaluating 18 different ROIs of 5 H&N patients imaged with CT before and during treatment. They found that all DIR strategies corresponded to improved DSC scores when compared with rigid registration (*p*<0.05, K-W test). Rigaud *et al*. [36] validated 10 different combinations of demons-based and fast free-form-based DIRs with weekly CT images in 15 H&N cancer patients, and they observed that the DSC scores of all DIR methods were remarkably superior to that of rigid registration (*p*<0.01, ANOVA). Mohamed *et al*. [16] acquired a similar result by utilizing 2720 anatomic ROIs and 50 tumor/nodal ROIs on CT images; they concluded that the 4 assessed DIR strategies (atlas-based, B-spline-based, demons-based and optical flow-based) were all improved over rigid registration (*p*<0.008 after Bonferroni correction, Steel test).

Interestingly, in contrast to these pilot findings, we found in in our study that rigid registration was not always inferior to DIRs (in terms of DSC) as one might expect, especially for low-contrast regions such as the SMG where rigid registration outperformed all the DIRs. This might be attributed to the fact that DIRs are more susceptible in the low-contrast SMG, which tends to fail in providing sufficiently contrasting edges to correctly drive the intensity-based DIRs [48].

For an individual DIR algorithm, the registration performance may vary for different ROIs. For example, the performance of HSLK in the PG was comparable to that in the SMG (*p*>0.05 for DSC and PE) but worse than that in the VTB (*p*<0.001 for DSC and PE) and the VF (*p* = 0.025 for DSC and *p*<0.001 for PE), where HSLK performed better in the VTB than in the VF (*p* = 0.029 for DSC and *p*<0.001 for PE). In addition, registration performance between different DIR algorithms also changes with different ROIs. For instance, DISC performed better than HSLK in the VTB but worse in the SMG, while the performance was comparable for the VF and the PG. These findings are in agreement with results of Mohamed *et al*. [16], who found that the differences among the registration accuracies of different structures varies substantially.

The demons-based DIRs (except for the improved demons-based algorithm, the DISC) were found to perform the worst in this study, implying that they might not be suitable for CT-CBCT DIR, although the demons-based DIRs are renowned for their effectiveness and robustness in CT-CT registration [14, 16, 36]. Although CT and CBCT are reconstructed under the same physical principles, the CT-CBCT DIR considered in this study is essentially an inter-modality DIR problem. This poses a great challenge for DIR methods, especially the demons-based DIRs, because of the notorious image quality in CBCT. CBCT suffers more severe image artifacts (e.g., cupping, streak and motion artifacts) and larger scatter contaminations than spiral CT [49–51]. Such artifacts usually result in intensity inconsistency between CT and CBCT, which tends to violate the intensity-consistency assumption in intensity-based demons algorithms.

By staging the treatment course, lower DIR accuracy was found in soft-tissue structures as the treatment proceeded, implying that the DIR performance is influenced by the magnitude of volume loss. This finding coincides with the results of Huger *et al*. [52], who observed evident deteriorating DIR accuracy in as the course of treatment progressed. Moreover, the advantage of DIR over rigid registration was observed in the later stage (stage 3, the 11th-15th fraction) of treatment, when larger volume changes usually occurred. This agrees with previous studies that re-planning should be carried out after 30Gy (i.e., the 15th fraction) [31] or from the 5th or the 15th fraction [35] to account for potential organ deformation.

One limitation of our current work is the exclusion of anatomical landmarks or fiducial markers for geometric accuracy validation. The reasons are twofold: first, all the image data employed were retrospectively collected instead of purposely designed for DIR validation. Thus, all the H&N patients are free of fiducial marker implantation. Second, severe noise and image artifacts in CBCT prevent accurately identifying the anatomic landmarks. Furthermore, landmark migration and stability is a widely debated issue. Distance variations between landmarks within 2 mm (95% confidence) and significant changes in landmark position relative to the tumor over a few weeks were reported [53, 54]. Inaccurate landmark identification will potentially bias the evaluated results.

## Conclusions

In this study, we comprehensively compared the ability of OAR contour propagation by ten intensity-based DIR strategies in the context of CT-CBCT DIR using CT/CBCT images retrospectively collected from twenty-one NPC patients. The experimental results indicated that all DIRs evaluated in this work did not necessarily perform better than rigid registration. Generally, the DIR performed better for bony structures than for soft-tissue organs, and the DIR performance tended to vary for different ROIs with different degrees of deformation as the treatment proceeded. Currently, physician-drawn contours are regarded as the ground truths, and human intervention is necessary when a clinically trustworthy DIR algorithm is not yet available. Even DIR algorithms proven to be effective in large-scale clinical data, skipping the contour reviewing procedure remains unlikely in the future to avoid possible failures in rare circumstances. Based on these observations, careful examinations or necessary modifications should be carried out before accepting the auto-propagated contours derived using the DIR algorithms evaluated in this work, especially for automatic re-planning ART systems.
